# Outcomes following different upfront stem cell transplantation strategies for multiple myeloma: a statistical perspective on behalf of the Chronic Malignancies Working Party of the EBMT

**DOI:** 10.1038/s41409-025-02675-2

**Published:** 2025-07-30

**Authors:** Simona Iacobelli, Stefan Schönland, Linda Koster, Didier Blaise, Emma Nicholson, A. E. C. Broers, Patrice Chevallier, Péter Reményi, František Folber, John. G. Gribben, Erfan Nur, Neil Rabin, Matthew Collin, Tobias Gedde-Dahl, Katharine Bailey, Paul Ferguson, Matthias Stelljes, Adrian Bloor, Meral Beksac, Joanna Drozd-Sokolowska, Kavita Raj, Patrick J. Hayden, Ibrahim Yakoub-Agha, Donal P. McLornan, Nicolaus Kröger

**Affiliations:** 1 Sapienza University of Rome, Rome, Italy; 2 University Hospital of Heidelberg, Medical Department V, Heidelberg, Germany; 3 EBMT Leiden Study Unit, Leiden, the Netherlands; 4 Transplantation & Cellular Therapy, Department of Hematology, Institut Paoli Calmettes, Management Sport Cancer Lab, Luminy, Aix Marseille University, Marseille, France; 5 Royal Marsden Hospital, London, United Kingdom; 6 Erasmus MC Cancer Institute, Rotterdam, Netherlands; 7 CHU Nantes, Nantes, France; 8 Dél-pesti Centrumkórház, Budapest, Hungary; 9 University Hospital Brno, Brno, Czech Republic; 10 St. Bartholomew’s Hospital London, London, United Kingdom; 11 VU University Medical Center, Amsterdam, Netherlands; 12 Department of Haematology, University College London Hospitals NHS Trust, London, UK; 13 Newcastle upon Tyne Hospitals, Newcastle, United Kingdom; 14 Oslo University Hospital, Rikshospitalet, Oslo, Norway; 15 Kings College Hospital London, London, United Kingdom; 16 Birmingham Centre for Cellular Therapy and Transplant (BCCTT), Birmingham, United Kingdom; 17 University of Muenster, Muenster, Germany; 18 Christie Hospital Manchester, Manchester, United Kingdom; 19 Ankara Liv Hospital, Istinye University, Ankara, Turkey; 20 University Clinical Centre, Medical University of Warsaw, Warsaw, Poland; 21 Department of Haematology, Trinity College Dublin, St. James’s Hospital, Dublin, Ireland; 22 CHU de Lille, Univ Lille, INSERM U1286, Infinite, Lille, France; 23 University Hospital Eppendorf, Hamburg, Germany

**Keywords:** Stem-cell research, Myeloma

## Abstract

Multiple myeloma (MM) is a heterogenous malignant disease. Novel agents including bispecific antibodies and chimeric antigen receptor (CAR) T cells have improved response rates and patient outcome, but the majority of patients ultimately still relapse. High dose chemotherapy followed by autologous hematopoietic stem cell transplantation (auto-HCT) remains standard care of treatment for transplant-eligible patients. While single auto-HCT is commonly used, a planned tandem auto-HCT or auto-allo approach remains controversial, based on conflicting results from clinical trials. Here we compared the outcome of 24,936 MM patients aged between 20 and 65 years who underwent first auto-HCT during 2002–2015, reported to the EBMT registry, of whom 3683 and 878 got tandem auto-HCT and auto-allo-HCT respectively. We used non-standard statistical approaches to account for time-dependence of treatments and of their effects, including models with multiple timescales and dynamic prediction. Differences were reported by graphs of hazard functions, hazard ratios and conditional probabilities over time. For both OS and PFS, there was a limited but persistent advantage for the tandem auto-HCT group compared to single auto-HCT, and a clear advantage for the auto-allo-HCT group over both other strategies in the longer term, albeit at the cost of higher early mortality.

## Introduction

Marked advances have been observed across the therapeutic landscape for multiple myeloma (MM). Novel treatments like proteasome inhibitors, IMiDs and monoclonal antibodies have improved response rates and survival [1–4]. However, despite high numbers of complete remissions (CR) and achievement of MRD negativity, the vast majority of patients still ultimately relapse. More recently, the advent of bispecific antibodies and CAR-T cells has revolutionized treatment algorithms [5]. Pivotal questions remain regarding the optimal sequencing and combinatorial approaches and how indeed these agents are best utilized in those patients considered as transplant eligible [4, 6].

For over three decades, high dose melphalan followed by autologous hematopoietic cell transplantation (auto-HCT) has been a standard approach in transplant-eligible patients. However, with improved risk prognostication in newly diagnosed MM (NDMM), the achievement of deep and durable responses after induction, and the increasing availability of MRD assessment, the timing of auto-HCT, and perhaps even the need for it in all patients, are currently under debate. Upfront auto-HCT following optimized induction could be offered to higher risk MM patients [7]. In resource constrained countries with limited access to novel agents and enhanced prognostication testing/MRD assessment, high dose melphalan is likely to remain standard-of-care for the foreseeable future.

In recent decades, the role of planned tandem HCT - either tandem auto-HCT or auto-HCT followed by allogeneic (auto-allo-HCT) - approaches in MM has remained controversial, based on conflicting results from clinical trials [8, 9].

Several studies investigated tandem auto-HCT to improve outcome after stem cell transplantation, but results are not conclusive [10–12]. The International Myeloma Foundation found a survival benefit after tandem auto-HCT only in patients not achieving at least a very good partial remission after the 1^st^ auto-HCT. The Italian Bologna 96 study [13] reported superior CR rates and event-free survival (EFS) after tandem transplantation but failed to demonstrate a prolonged overall survival (OS). In the German GMMG-HD2 trial [14], tandem transplantation increased the number of responses but did not result in either prolonged EFS or OS. The EMN02/HO95 study [15] however found significantly improved OS and EFS for patients who received tandem auto-HCT in the overall cohort, especially in high-risk patients.

Harnessing a potential graft versus myeloma effect with an allo-HCT in eligible patients was considered attractive though often resulted in considerable early toxicity and non-relapse mortality (NRM). However, over time, improved donor selection and availability, enhanced supportive therapy and the use of non-myeloablative conditioning regimens have led to decreases in NRM [16]. The EBMT NMAM2000 study prospectively compared tandem auto-allo-HCT, based on availability of an HLA-matched sibling donor, to auto-HCT alone or tandem auto-HCT at the discretion of the center. Longer term outcome, with regard to eight year OS and progression free survival (PFS) rates, was improved for patients in the auto-allo-HCT cohort compared to auto-HCT alone, clearly if they survived the early risk of NRM [17, 18]. The BMT CTN 0102 trial compared tandem auto-HCT with auto-allo-HCT in NDMM patients with either standard or high-risk disease [8, 9]. Longer term analysis, at a median follow up of ten-years, found that the auto-allo-HCT approach conferred a significant reduction in the six-year risk of relapse in the high-risk NDMM group and a trend to improved PFS and similar OS for those undergoing the auto-allo-HCT approach compared to tandem auto-HCT. Costa and colleagues [19] analyzed patient data from four trials comparing tandem auto-HCT with auto-allo-HCT, and their meta-analyses demonstrated improved longer term PFS, OS and post-relapse survival in the auto-allo-HCT cohort. However, this was only achieved with considerable NRM, which was higher in the auto-allo-HCT cohort compared to the tandem auto-HCT cohort. Overall, despite these positive results, an auto-allo-HCT tandem approach has not become standard of care in treatment of younger high-risk MM patients due to risks of GvHD and NRM and the emergence of the above-mentioned new treatment modalities.

This retrospective registry-based study performed on behalf of the Chronic Malignancies Working Party (CMWP) of the EBMT evaluated outcomes following upfront single auto-HCT, tandem auto-HCT and planned auto-allo-HCT in 24,936 MM patients, the largest such analysis to date with long follow up, making use of sophisticated statistical approaches for clinically relevant analyses. Our data and proposed statistical methods may be useful for further outcome comparison with immunotherapeutic approaches including CAR-T cells and bispecific antibodies which are now included in first-line therapy trials, but with real-world data only existing in later lines.

## Methods

**Fig. 1:**
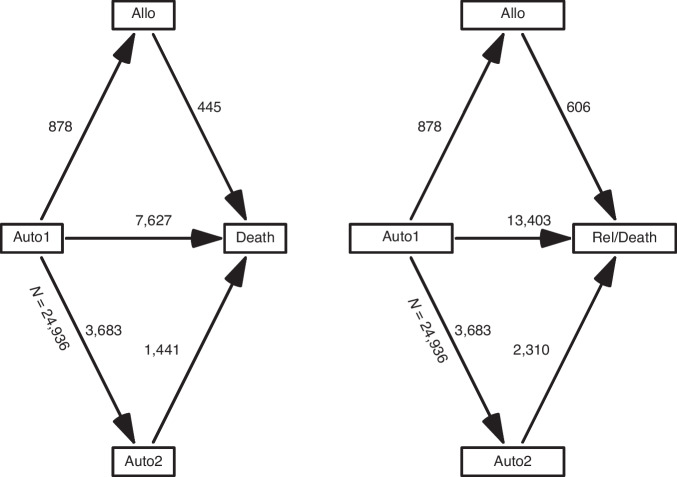
Schemas representing the disease histories of interest as a Multi-State Models with 4 states, of which 1 absorbing, and 5 transitions. Left: Overall Survival (OS). Right: Progression-Free Survival (PFS) (see Supplementary Material).

Tandem strategies were defined as a second transplant occurring within nine months of the first auto-HCT (auto-HCT1) in the absence of a prior relapse or disease progression. Endpoints were OS and PFS, defined as time from auto-HCT1 to death and to progression or death, respectively. In the EBMT registry, we initially evaluated for data quality a cohort of 47,746 MM patients aged between 20 and 65 years who underwent auto-HCT1 between 2002 and 2015. We decided to exclude all cases from centers where completeness of follow-up information was suboptimal for more than 5% of their patients. Slightly higher OS and PFS of cases excluded confirmed that these centers may have missed reporting events for a proportion of their patients. The final population included 24,936 patients. The histories analyzed are illustrated as multi-state models in Fig. 1. The data presented two challenging features. One lies in the fact that treatment strategies (administration or not, and type of, second transplant) were not fixed on an intent-to-treat basis, and groups being compared were established after the time origin of the endpoints (the day of auto-HCT1). Hence, appropriate methodology has to be employed to avoid an “immortal” time bias [20]. The other feature of relevance is that the impact of the second transplant, in particular of allo-HCT, depends on the time since its occurrence (for example, the risk of death is much higher in the first six months following allo-HCT than after two years; Supplementary Fig. S1). This requires incorporation of this second timescale into the models, while the usual survival analysis methods use only one timescale (in our study, time since auto-HCT1). We used multiple statistical approaches, including Poisson regression with multiple timescales and dynamic prediction. A thorough description of the methodology employed is provided in the statistical section below and in the Supplementary Material.

The study was planned and approved by the CMWP of the EBMT. EBMT centers are committed to obtain informed consent according to the local regulations applicable at the time of transplantation in order to report pseudonymized data to the EBMT. The study was conducted in accordance with the Declaration of Helsinki and Good Clinical Practice guidelines.

### Statistical methods

Standard landmark curves were utilized for the initial illustration of outcomes based on the transplantation strategy. We next analyzed the hazard functions applying two alternative approaches to account for the dependence of the post-tandem transplant hazard on the time since its administration. One was Cox regression with tandem transplants included as time-dependent covariates, with time-varying effects modeled by constant hazard ratios (HR) in four periods: the first six months, from six to twelve months, from twelve to 24 months, and after 24 months from administration. The second approach was a parametric Poisson regression allowing the baseline hazard to flexibly depend both on time since auto-HCT1 and, for tandem strategies, on the time since the second transplant [21]. We showed that these two approaches gave consistent results (Supplement). To quantitate the differences in terms of OS and PFS probabilities we computed dynamic prediction curves using the “landmarking” approach [22, 23]. Additional explanations and details are provided in the Supplementary Material.

## Results

**Fig. 2:**
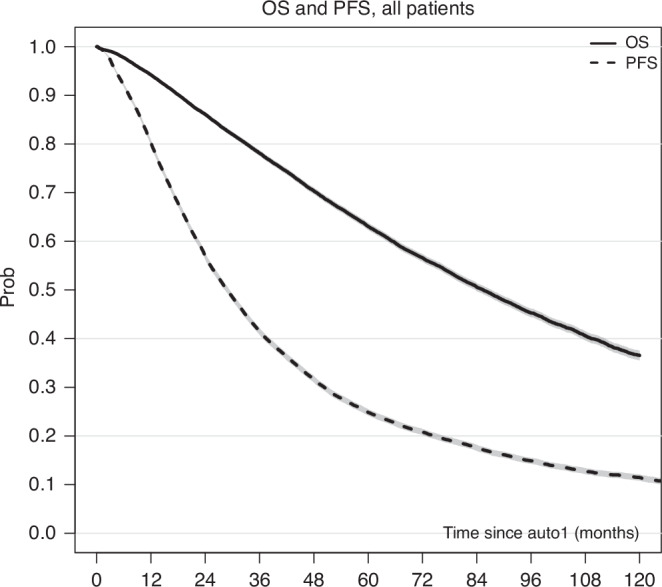
Overall survival and progression-free survival. Overall survival (OS, solid line) and progression-free survival (PFS, dashed line) Kaplan–Meier estimates, with bands for the 95% CI at each time point, for the whole study population (*n* = 24,936).

Out of 24,936 patients, 20,375 patients underwent single auto-HCT, 3683 underwent tandem auto-HCT and 878 underwent an auto-allo-HCT approach (Fig. 1). For the entire study population, at a median follow-up of 66.3 months, the median OS was 86 months, with an 8-year OS probability of 45.3% (95% CI: 44.4–46.1). The median PFS was 29 months, and the three-year PFS was 41.5% (95% CI: 40.9–42.2; Fig. 2). The cumulative incidence of administration of a tandem transplant was 15.6% for tandem auto-HCT and 3.7% for auto-allo-HCT, with median timings of 3.6 and 3.9 months, respectively. Patient, transplant details and disease status for the overall cohort and the three groups are displayed in Table 1. Patients who underwent auto-allo-HCT tended to belong to earlier calendar year cohorts (only 10% were transplanted between 2012 and 2015 compared to 15% for tandem auto-HCT and 24% for single auto-HCT), to be younger (42% were <50 years-old and 11% >60 years-old compared to 20% and 32%, respectively, in the other groups) and had worse disease status at auto-HCT1 (CR rate was 8%, similar to tandem auto-HCT but lower than single auto-HCT (20%)).

**Table 1:** Characteristics of patients grouped by transplant strategy given.

		Single auto-HCT		Tandem auto-HCT		Auto-allo-HCT		All cases	
		Frequency	%	Frequency	%	Frequency	%	Frequency	%
Sex	Male	11,670	57.3%	2215	60.1%	554	63.1%	14,439	57.9%
	Female	8705	42.7%	1468	39.9%	324	36.9%	10,497	42.1%
	Total	20,375	100.0%	3683	100.0%	878	100.0%	24,936	100.0%
Age at first auto	18–50	3943	19.4%	769	20.9%	369	42.0%	5081	20.4%
	50–60	9782	48.0%	1791	48.6%	411	46.8%	11,984	48.1%
	60–65	6650	32.6%	1123	30.5%	98	11.2%	7871	31.6%
	Total	20,375	100.0%	3683	100.0%	878	100.0%	24,936	100.0%
	Median (Q1,Q3)	57.2 (51.7, 61.2)		56.7 (51.2, 60.9)		51.7 (45.5, 56.9)		57.0 (51.4, 61.0)	
Year of first auto	2002–2006	5863	28.8%	1826	49.6%	491	55.9%	8180	32.8%
	2007–2011	9705	47.6%	1299	35.3%	302	34.4%	11,306	45.3%
	2012–2015	4807	23.6%	558	15.2%	85	9.7%	5450	21.9%
	Total	20,375	100.0%	3683	100.0%	878	100.0%	24,936	100.0%
Time diag- first auto	<12mo	15,696	77.0%	2983	81.0%	754	85.9%	19,433	77.9%
	>=12	4679	23.0%	700	19.0%	124	14.1%	5503	22.1%
	Total	20,375	100.0%	3683	100.0%	878	100.0%	24,936	100.0%
Status at first auto	CR	4015	19.7%	339	9.2%	71	8.1%	4425	17.7%
	PR	13,923	68.3%	2651	72.0%	590	67.2%	17,164	68.8%
	Other/NA	2437	12.0%	693	18.8%	217	24.7%	3347	13.4%
	Total	20,375	100.0%	3683	100.0%	878	100.0%	24,936	100.0%
MM classification	Ig G	10,463	52.7%	1909	53.7%	452	52.6%	12,824	52.9%
	Ig A	3739	18.8%	571	16.1%	178	20.7%	4488	18.5%
	Light chain	4829	24.3%	945	26.6%	192	22.3%	5966	24.6%
	Other	820	4.1%	128	3.6%	38	4.4%	986	4.1%
	Total	19,851	100.0%	3553	100.0%	860	100.0%	24,264	100.0%
Time diag- first auto	<12mo	15,696	77.0%	2983	81.0%	754	85.9%	19,433	77.9%
	>=12	4679	23.0%	700	19.0%	124	14.1%	5503	22.1%
	Total	20,375	100.0%	3683	100.0%	878	100.0%	24,936	100.0%
Chain type	Kappa	11,556	64.9%	2145	67.2%	529	65.6%	14,230	65.3%
	Lambda	6190	34.8%	1035	32.4%	278	34.4%	7503	34.4%
	Both kappa and lambda	50	0.3%	10	0.3%	0	0.0%	60	0.3%
	Total	17,796	100.0%	3190	100.0%	807	100.0%	21,793	100.0%

Significance testing for differences between the 3 groups is irrelevant, being warranted by the very large sample size (all *P*-values < 0.001, not displayed). Auto-allo-HCT were RIC in 85% of the cases; donors were Hla-identical sibling (72%), matched unrelated donor (25%) or other (3%).

**Fig. 3:**
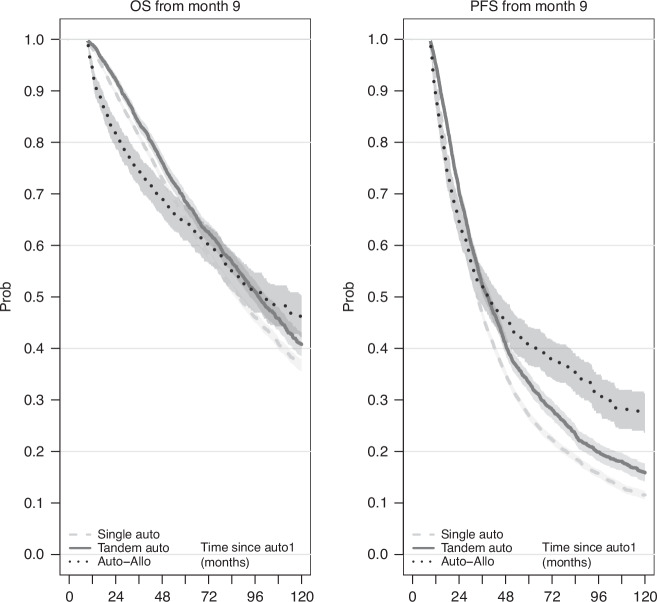
Landmark Kaplan–Meier curves, from 9 months after first auto-HCT. Left: Conditional overall survival. Right: Conditional progression-free survival. The landmark time was set at 9 months, being the maximum time of administration of a tandem transplantation approach.

**Fig. 4:**
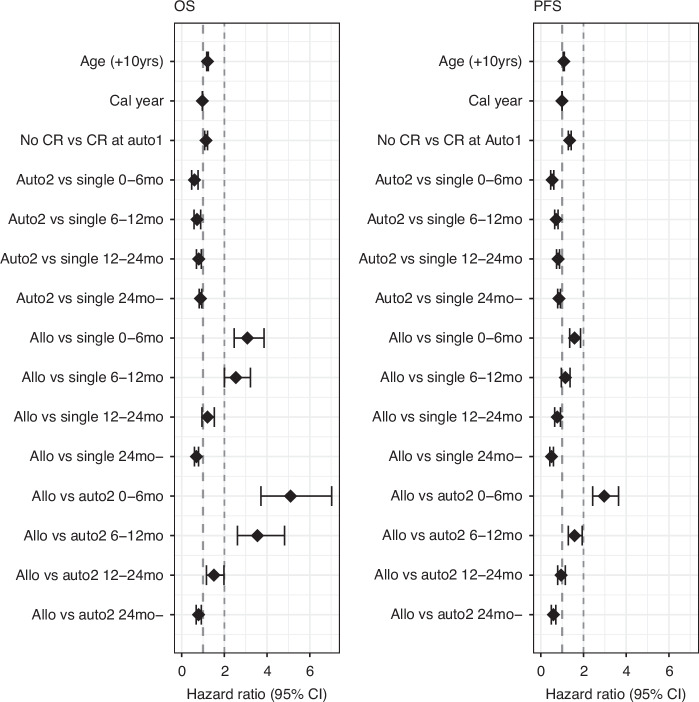
Cox models, estimated Hazard Ratios (HR) with 95% CI. Forest plots; the corresponding estimates are displayed in Supplementary Table S1. Effects of tandem transplants split in time periods since their administration. These are also shown in Supplementary Fig. S3 for comparison with the Poisson models. Adjustment for characteristics measured at first auto-HCT; Age and Calendar Year as continuous variables, HRs quantifying the effect of +10 years of age and of +1 calendar year respectively; Disease Status dichotomized as not being in Complete Remission (CR) versus being in CR. 95% CI not including the value 1 indicates significance at 5% level.

Figure 3 provides a first illustration of the OS and PFS of the three transplant strategy groups based on a landmark analysis. We used two alternative approaches to model the hazards according to the administered transplant strategy, adjusting for characteristics at auto-HCT1, and accounting for the variation of the impact of the administration of a tandem transplant on the hazard along the time since administration, further than on the time since auto-HCT1 (which is the only timescale defined for the single auto-HCT group). Utilizing Cox regression, we estimated the differences between tandem transplant strategies and single auto-HCT (baseline) by simple HR in four periods measured since the administration of the second transplant (Fig. 4 and Supplementary Table S1). The change associated with the tandem auto-HCT was a statistically significant reduction of instantaneous risk in every interval, which decreases over time since its administration (OS: HR from 0.6 to 0.88 from first to last period; PFS: from 0.53 to 0.85). The change associated with the auto-allo-HCT consisted of an initially increased risk (OS: HR = 3.08 and HR = 2.54 in 0–6-months and 6–12 months; PFS: HR = 1.58 in 0–6 months), later reversed with a significant protection in the longer term (OS: HR = 0.69; PFS: HR = 0.5 i.e., risk halved after two years from administration of allo-HCT).

**Fig. 5:**
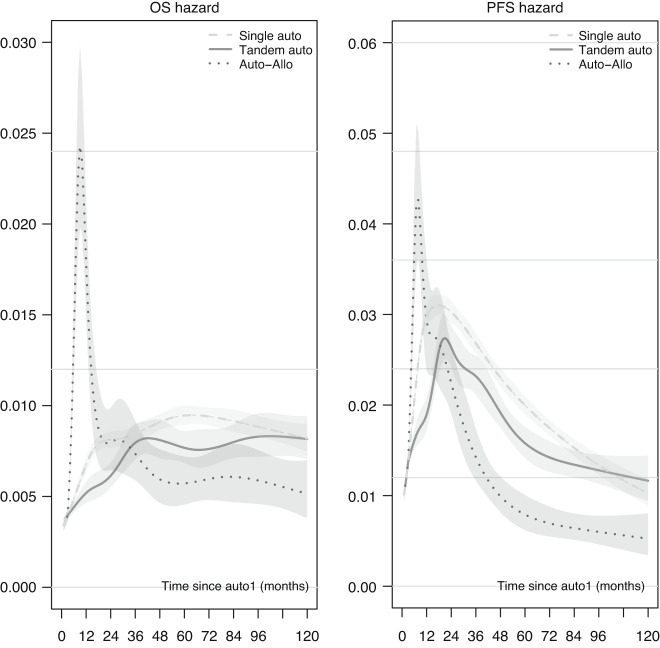
Hazard functions for Single auto-HCT, tandem auto-HCT and tandem auto-allo-HCT estimated using the Poisson model with two time scales. The bands correspond to the limits of 95% CI. Pattern of characteristics at first auto: Age = 55, Calendar year = 2008, Disease Status = no Complete Remission. Timing of tandem transplant: 3 months after first auto-HCT.

These results were confirmed by a second approach to MVA, whereby we used a two time-scales Poisson regression, obtaining hazard and HR estimates in continuous time, as highlighted in Fig. 5 and Supplementary Fig. S3 (showing also the consistency with Cox results). It is evident that there was a limited but persistent advantage for the tandem auto-HCT group compared to single auto-HCT for both OS and PFS. Regarding auto-allo-HCT, there was a clear advantage in the longer term over both other strategies, but at the cost of a very high peak of risk in the first few months after allo-HCT. With both MVA approaches, the effects associated with the characteristics at auto-HCT1 were as follows (Supplementary Tables S1 and S2); older age increased the risk of death (+21% for each additional ten years) and of progression or death (+8%). Both OS and PFS improved with calendar time (−3% and −1% instantaneous risk per year respectively). Not being in CR at the time of auto-HCT1 compared to being in CR increased the OS risk by 14% and the PFS risk by 35%.

### Conditional OS and PFS probabilities

**Fig. 6:**
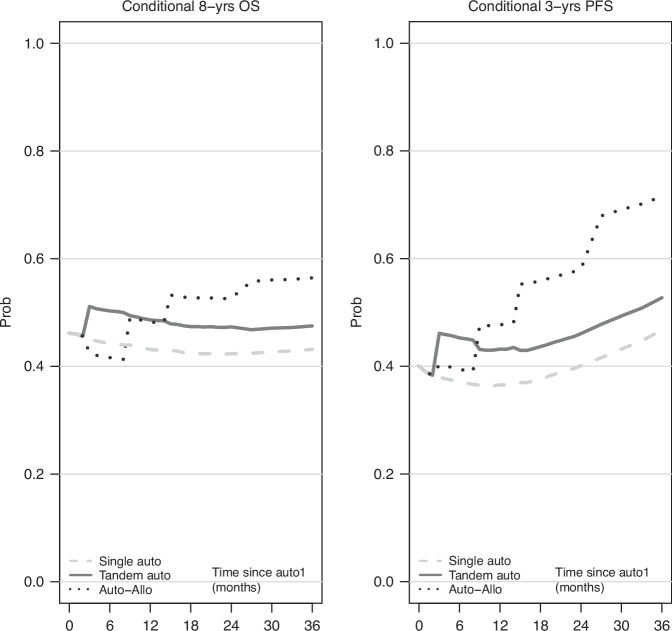
Dynamic prediction curves. Left: Conditional 8-years overall survival. Right: Conditional 3-years progression-free survival. Pattern of characteristics at first auto-HCT: Age = 55, Calendar year = 2008, Disease status = no complete remission. Timing of tandem transplant: 3-months after first auto-HCT. For example: For patients alive 36-months after first auto-HCT, the probability of being still alive 8-years later was 43.2% for single auto-HCT, 47.5% for tandem auto-HCT, and 56.4% for tandem auto-allo-HCT. For patients’ alive relapse/progression-free 36-months after first auto-HCT, the probability of being still alive relapse/progression-free after additional 3-years was 46.8% for single auto-HCT, 52.7% for tandem auto-HCT, and 71.5% for tandem auto-allo-HCT.

In view of the dynamic change in risk seen with tandem auto-allo-HCT from being associated with more risk at the start to relative protection in the longer term, it was important to evaluate the global impact in terms of survival probabilities. We utilized the same model structure of the previous Cox regression analyses to implement the method of dynamic prediction by landmarking (Supplementary Material) for estimation of the conditional eight-year OS and the three-year PFS probabilities for patients surviving (progression-free, for the latter) during the first three years after auto-HCT1 (Fig. 6). The projected probability of long-term survival (here, at eight years) was, overall, relatively stable with any of the three transplantation approaches, however, with a slight improvement only for patients who received a tandem auto-allo-HCT. Looking at the end of the prediction period, the advantage with tandem auto-allo-HCT in terms of eight-year OS probability was quantified by +9% versus tandem auto-HCT and +13% versus single auto-HCT. The chance of remaining alive in a progression-free state for the next three years improved in those who had survived progression-free for at least two years, modestly with single and tandem auto-HCT and again more markedly with tandem auto-allo-HCT, which at the end of the period had a 19% advantage when compared to tandem auto-HCT and a 25% advantage compared to single auto-HCT.

## Discussion

This study represents one of the largest assessments to date of the comparison of tandem auto-HCT or auto-allo-HCT and single auto-HCT in MM. The potential long-term benefit of an auto-allo-HCT approach remains controversial given the higher rates of early NRM in allo-HCT, and it has not become a standard approach, even for potentially eligible patients with high-risk disease [24]. Wei and colleagues performed a comparative meta-analysis of three studies including 491 patients with high-risk MM [25]. An auto-allo-HCT approach was associated with improved PFS and a higher rate of CR though there was no significant effect on OS. Gagelmann et al. reported on 488 high-risk MM patients with extramedullary disease (EMD) who underwent either a single auto-HCT, tandem auto-HCT or tandem auto-allo-HCT [26]. A tandem auto-HCT approach in high-risk cytogenetic MM patients with EMD resulted in a four-year PFS of 45% compared to 22% with single auto-HCT suggesting better disease control with tandem auto-HCT. Conclusions on the exact place of auto-allo-HCT in this setting, however, could not be established given the small numbers (*n* = 31). Kroger and colleagues recently published on 178 MM patients from 20 German centers enrolled in an open-label trial (2008-2014) which compared tandem auto-HCT (*n* = 46) and auto-allo-HCT (*n* = 132) followed by two years of thalidomide maintenance [27]. Tandem auto-allo-HCT resulted in a reduction in MM progression or relapse by 23% at four years and 33% at eight years but the improvement in PFS failed to reach statistical significance, probably due to the low number of tandem-auto-HCT. Long term OS did not differ between the cohorts.

The EBMT registry data shows that more clinicians were inclined to offer the auto-allo-HCT approach in earlier calendar years, in younger patients and in those with a worse disease status at the time of auto-HCT1. Patients of this cohort surviving through the high risk of NRM during the early months following allo-HCT had a long-term advantage compared to the other cohorts, more limited for OS and more marked for PFS. We also identified a small though persistent advantage for both PFS and OS with tandem auto-HCT compared to single auto-HCT. We quantified the differences not only as HR, but also in terms of probabilities, estimating conditional OS and PFS (dynamic prediction curves).

Our results are in line with the existing literature. Importantly, our data regard the general population, opposite to clinical trial studies. We have enhanced the registry data, extracting information such as the conditional probabilities, which can be used for the discussion with patients who have survived the transplant to inform him/her about the expected outcomes. E.g., for auto-allo, three years after first auto-HCT the probability of surviving without progression for 3 additional years is 70%. Regarding OS, there is a 60% probability of surviving for the next 8 years. Interestingly, these probabilities are quite high, reinforcing the concept of cure for first-line therapy in MM.

Our study has the important limitation that the groups could not be defined on an intent-to-treat basis but were the result of “natural selection”, with patients experiencing early relapse/progression or death having less chances of proceeding to a tandem transplant approach, and therefore being more likely to belong to the single auto-HCT group. This mechanism has anyway a limited impact when comparing groups conditionally in the long-term, such as when looking at the hazard functions (Fig. 5) or at the dynamic prediction curves (Fig. 6). As with any retrospective study, we could only partially control for indication bias i.e., for different characteristics leading to the choice of a given transplantation strategy. However, in the MVA, we have considered gender, age, disease status at first auto-HCT, calendar year and interval from diagnosis to auto-HCT1. Our analytical approach carefully considered the problem of defining the comparisons groups after the start of the follow-up time, by using traditional and more advanced statistical methods. We demonstrated that when comparing a strategy including allo-HCT the usual Proportional Hazards assumption is strongly violated (Supplementary Fig. S1), and the modeling should consider the use of time since allo-HCT as a second timescale. Similarly, any comparison between treatment strategies characterized by a ‘trade-off’ between short-term safety and long-term efficacy faces the same difficulty and needs to be addressed. We here show two alternative approaches, one using Cox regression with piecewise-constant time-varying effects for time-dependent covariates, the other based on a flexible Poisson model that can easily include multiple timescales. Such a situation is not infrequent in clinical studies, and demography and epidemiology have a long tradition of using methods such as including both current age and calendar time in the calculation of event rates [28–31]. We suggest considering these timescales in any study evaluating longer-term outcomes, and when investigating time-scale issues with any multi-state data [32]. We proposed a Cox-based approach, which produces familiar output (HRs with 95% CI and significance test) though returning only an average effect over pre-specified time periods; and a Poisson-based approach, which overcomes the rigidity of the other one, and allows visualization of the effects of all timescales in terms of hazards or HRs (with 95% CI). The last methodological challenge of evaluating how hazard ratios of time-dependent covariates varying in time impact on survival probabilities was addressed by using a recent method for dynamic prediction curves, which incorporates the complexities of multi-state data, still using familiar concepts as the landmark analysis.

In conclusion, we could show and quantify improved long-term outcome in a very large dataset of MM patients who received tandem auto-HCT or auto-allo-HCT in comparison to single transplantation. The proposed statistical methods allow to correctly compare outcomes in complex diseases, where several lines of treatment are given during the course of the disease, and can be useful for future studies comparing other time dependent strategies such as CAR-T and bispecific antibodies.

## Supplementary information

Supplementary Material

## Data Availability

The final analysis dataset will be available upon specific request to the Working Party chair.
