# Supplementary material for: Outcomes following different upfront stem cell transplantation strategies for multiple myeloma: a statistical perspective on behalf of the Chronic Malignancies Working Party of the EBMT
Source: Bone Marrow Transplant. 2025 Jul 30;60(10):1361–8. doi: 10.1038/s41409-025-02675-2 (PMC12568642; doi:10.1038/s41409-025-02675-2)
Supplement: Supplementary file 1 — Supplementary Material [file 41409_2025_2675_MOESM1_ESM.pdf]

Supplementary Material

Contents

Disease-histories as multi-state models.. 1
Investigating the impact of type of tandem transplant... 2
Modelling hazards, approach 1: Cox regression 3
Modelling hazards, approach 2: Poisson regression... 5
Estimating probabilities: Dynamic prediction curves 8
Multi-variable modelling details.. 9

Disease-histories as multi-state models

Figure 1 represents the core elements of the disease histories investigated in our study as multi-state models, i.e. as patterns of transitions (the arrows in the graphs) through a series of relevant states (the boxes). Several statistical approaches can be used to analyze a multi-state model[1].The choice depends on the objective and on certain specific features of the data. For example, the estimation of all transition hazards and corresponding probabilities is rather straightforward when all transitions' risks satisfy the Markov property, i.e. the risk of making a transition from state A to a state B does not depend neither on time when A was entered, nor on time since A was entered[2]. We aimed to illustrate the impact of the administration of (type of) tandem transplant both on the hazard of event, and on the long-term event-free survival probabilities. As shown in the next section, we could not assume the validity of the Markov property.

Investigating the impact of type of tandem transplant

Figure S1 illustrates nonparametric smoothed estimates of the hazard functions for tandem second transplant, either auto-HCT or allo-HCT[3]. There is clear evidence that these hazards are non-proportional (i.e. time-varying) and that they must be modelled along the timescale that starts at time of 2nd transplant episode.

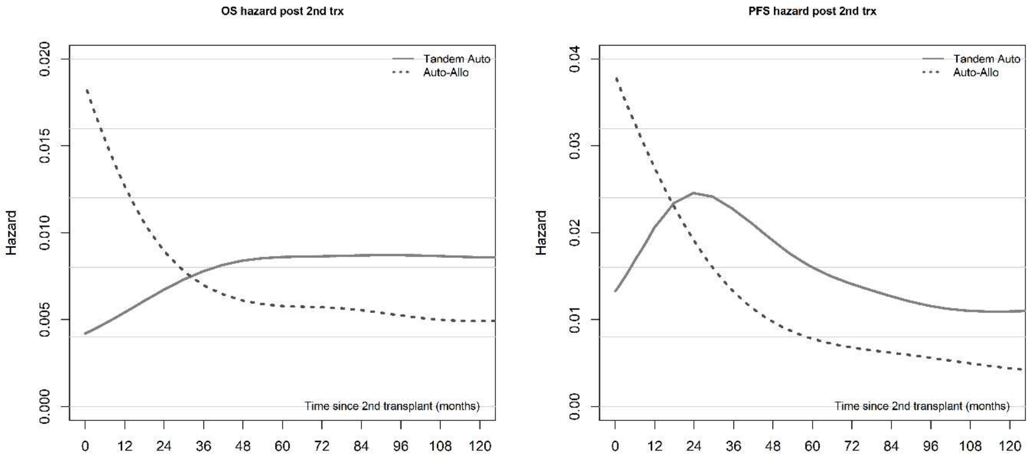

Figure S1. Hazard functions after 2nd transplant, non-parametric estimates. Left: OS. Right: PFS.

For completeness, we show also the corresponding Kaplan-Meier curves. It is in fact important to visualize how the benefit in terms of hazard of one strategy compared to the other translates in terms of actual event-free survival probability.

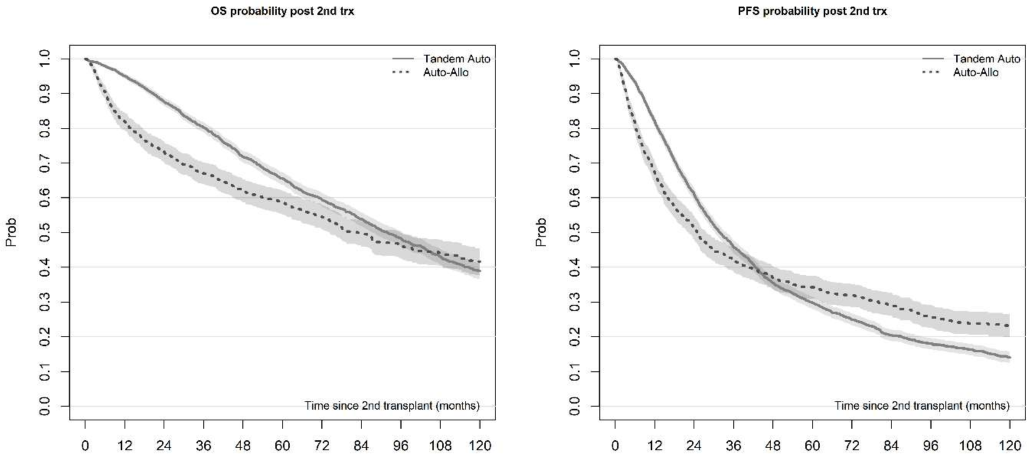

Figure S2. Kaplan-Meier curves after second transplant. Left: OS. Right: PFS. The bands correspond to the 95%CI at each time point. The difference Allo-Auto at 10 years was 2.7% (95%CI: -1.9% ,+7.2%) for OS and 9.2% (95%CI: +5.5% ,+12.9%) for PFS.

Modelling hazards, approach 1: Cox regression

Cox regression can be used to model the transition hazards of a multi-state disease history typically by incorporating the occurrence of intermediate events (entry into the intermediate states) as time-dependent covariates. While the assumption at the basis of the model is that hazards are proportional (PH), as we saw in the previous section in our study, tandem transplants had instead time-varying effects, which depend on time since 2nd transplant. We thus included in the Cox model the effect of tandem transplants as piecewise constant on four time intervals measured along time since second transplant: 0-6 months, 6-12 months, 12-24 months and after 24 months.

The relevant time split was anticipated based on substantive knowledge, and confirmed based on the observed data; specifically we evaluated the variation of the hazard functions (**Figure S1**) and we checked that each period included a sufficient number of events to allow the estimation of the effect in that period (for PFS further split of the period >24 months would have been useful, but we preferred to keep the model simple, also for keeping a minimal number of events in some period for the smaller auto-allo-HCT group). Since we aimed to applying dynamic prediction for computing long-term OS and PFS probabilities, for consistency between the two analyses, in estimating the Cox models we used only the follow-up information respectively of the first 11 years for OS and 6 years for PFS (being 11 and 6 equal to the sum of the length of prediction interval, 3 years, plus the length of the prediction horizon, 8 and 3 years respectively: see the dedicated supplementary section). The results of Cox models are displayed in **Figure 4** and **Table S1.**

	OS				PFS			
	HR	95%CI		p-value	HR	95%CI		p-value
**Age (+10yr)**	1.21	1.17	1.25	<0.001	1.08	1.05	1.10	<0.001
**Year (+1yr)**	0.97	0.97	0.98	<0.001	0.99	0.99	1.00	0.002
**Status: no CR vs CR**	1.14	1.08	1.21	<0.001	1.35	1.29	1.42	<0.001
**Tandem Auto vs Single Auto:**								
**Period:0-6mo**	0.60	0.47	0.77	<0.001	0.53	0.46	0.61	<0.001
**Period:6-12mo**	0.72	0.58	0.89	0.002	0.72	0.65	0.81	<0.001
**Period:12-24mo**	0.80	0.69	0.92	0.002	0.81	0.74	0.88	<0.001
**Period:>24mo**	0.88	0.82	0.94	<0.001	0.85	0.79	0.92	<0.001
**Allo vs Single Auto:**				<0.001				<0.001
**Period:0-6mo**	3.08	2.46	3.86	<0.001	1.58	1.35	1.86	<0.001
**Period:6-12mo**	2.54	2.00	3.22	<0.001	1.15	0.96	1.37	0.137
**Period:12-24mo**	1.21	0.95	1.53	0.121	0.77	0.65	0.92	0.003
**Period:>24mo**	0.69	0.60	0.79	<0.001	0.50	0.42	0.59	<0.001
**Allo vs Tandem Auto:**								
**Period:0-6mo**	5.10	3.71	7.03	0.000	2.97	2.43	3.64	0.000
**Period:6-12mo**	3.55	2.61	4.82	0.000	1.58	1.29	1.94	0.000
**Period:12-24mo**	1.51	1.16	1.98	0.002	0.95	0.79	1.15	0.605
**Period:>24mo**	0.79	0.68	0.92	0.002	0.59	0.49	0.70	0.000

Table S1. Comparisons between transplant strategies. Cox model estimates. Adjustment factors were Age, Calendar Year and Disease Status at first auto. The effect of tandem transplant (tandem auto-HCT or auto-allo-HCT) is split along time elapsed since administration of second transplant, as explained above (**Figures 4** and **S3**).

Modelling hazards, approach 2: Poisson regression

Our approach based on Cox regression had both advantages and limitations. The advantages lie in the simplicity (it was possible to use standard software for Cox regression in the counting processes formulation, applicable after basic data preparation steps) and ease of interpretation (comparisons of transplant strategies were done by HRs). Furthermore, it was possible using the same modeling for obtaining dynamic prediction curves, as discussed below, in a relatively straightforward way. The limitations were the rigidity of the structure with piecewise constant HRs for the impacts of tandem transplants, and the dependence of results on the cut-points chosen for the time split. Using more flexible functions of time was of course possible, but at the cost of losing the advantages just described. As an alternative approach, which could be considered an extension with a more flexible structure, we applied a Poisson regression with parametric baseline hazard allowed to depend on multiple timescales, obtaining the graphs of the hazard functions and HRs illustrated in **Figures 5** and **S3**.

Specifically, we considered a PH model of the type described by Efron (2002), with baseline hazard given by the product of three components, depending respectively on time since first auto-HCT (main timescale), time since tandem auto-HCT and time since allo-HCT (the latter two components applicable only for the hazard after tandem transplant in the two groups)[4]. We modelled the baseline hazards using restricted cubic splines with 6 knots (5 parameters for each spline). The cut-points were fixed based on quantiles of time-to-event observed overall (first component) and in the two tandem groups (the other components), such that in each interval there was the same number of events. The tests for the dependence of the hazard on time since allo-HCT was highly significant for both OS and PFS ($p<0.001$), while the dependence of the hazard on time since second auto-HCT was non-significant ($p=0.17$ for OS and $p=0.80$ for PFS), confirming that the effect associated to tandem auto-HCT was substantially constant or only slightly decreasing in time, as seen in the Cox-based analysis. More generally, as shown in **Figure S3** our "rigid" Cox model approximates in an acceptable fashion the variation of the hazard ratios as estimated by the Poisson regression. Also the effects of the adjustment covariates estimated in the Poisson model (**Table S2)** were very similar to those estimated with Cox regression.

The similarity of results between the two approaches is not unexpected, it is in fact well-known that a PH model with constant baseline hazard in each of a sequence of small time intervals and a Poisson model for independent counts for events along the same sequence of intervals with the log of the interval length included as offset have equivalent likelihoods[5].

The advantages of the Poisson approach lie in the powerful graphical representations of hazards and HRs at the cost of a relatively easy implementation of the estimation even in the presence of multiple relevant timescales and using flexible functions to model their effects. In fact, the estimation is obtained using standard software for

GLM (Generalized Linear Models) after a preliminary preparation of a suitable dataset, with a fine time-split into small intervals (necessary as said for the equivalence of a Poisson likelihood and a PH time-to-event likelihood) and with respect to the occurrence of tandem transplants. We used the tools for time-split available in the R package Epi[6]. The computational burden once cumbersome is nowadays affordable with current computers. An alternative approach uses flexible parametric survival models and provides very similar estimates[7].

	**OS**				**PFS**			
	**HR**	**95%CI**		**p-value**	**HR**	**95%CI**		**p-value**
**Age (+10yr)**	1.21	1.17	1.24	<0.001	1.07	1.05	1.10	<0.001
**Year (+1yr)**	0.97	0.97	0.98	<0.001	0.99	0.99	1.00	0.004
**Status: no CR vs CR**	1.14	1.08	1.21	<0.001	1.34	1.28	1.40	<0.001

Table S2. Poisson model estimates. Adjustment factors were Age, Calendar Year and Disease Status at first auto as in Cox regression. The effects of tandem transplants were incorporated in the baseline hazard; thus the approach does not lead to estimated HR for Tandem Auto or Auto-Allo vs Single Auto; the hazard and HR are shown in **Figures 5** and **S3**.

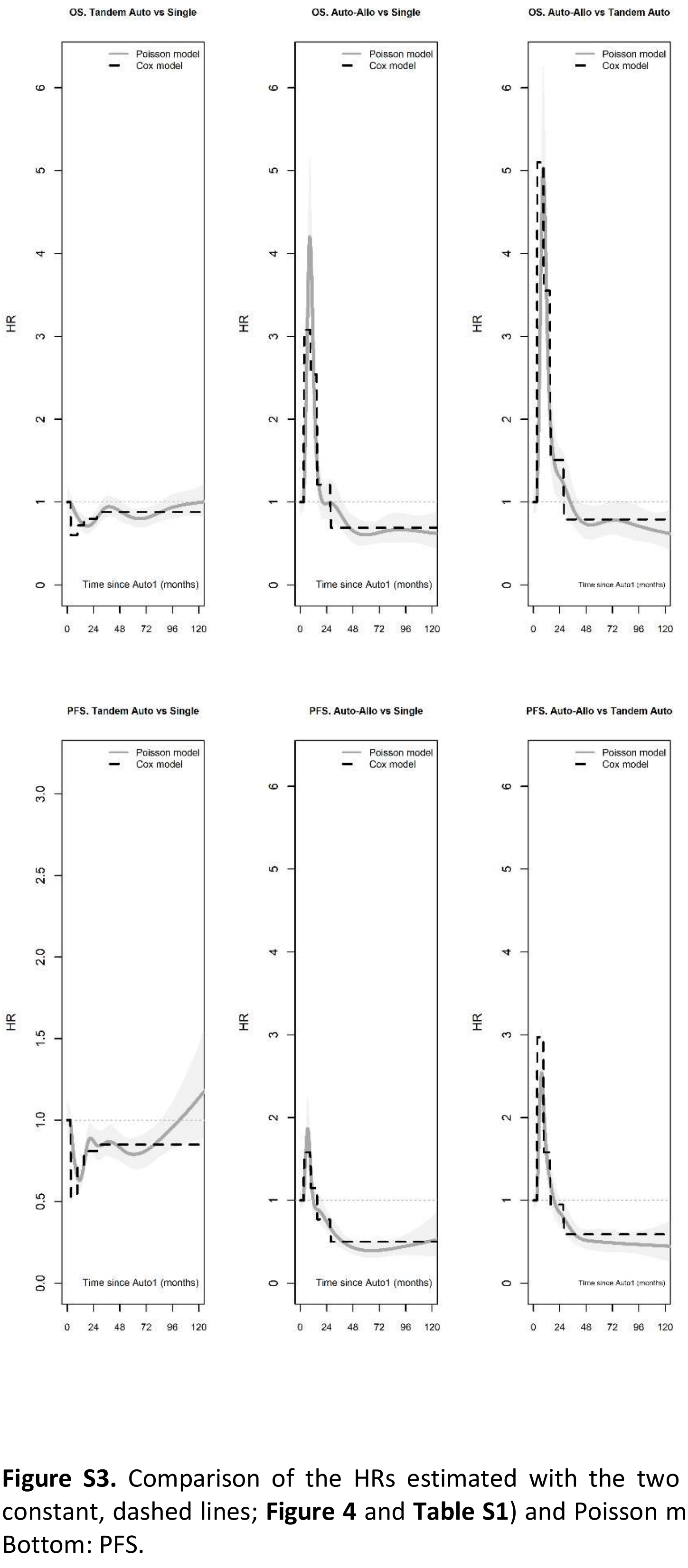

Figure S3. Comparison of the HRs estimated with the two regression approaches, Cox model (piecewise-constant, dashed lines; **Figure 4** and **Table S1**) and Poisson model (using two time scales, solid lines). Top: OS. Bottom: PFS.

Estimating probabilities: Dynamic prediction curves

Following evaluation of differences between transplant strategies as Hazard Ratios, it is important to evaluate also how these translate on the (progression-free) survival probabilities of the three groups. This is not straightforward in the presence of time-dependent covariates[8]; in particular, as said above, in our study the Markov property which is at the basis e.g. of the Simon-Makuck curves or of the Aalen-Johansen estimators, is strongly violated. The traditional landmark curves (as shown in **Figure 3**) are a correct but inefficient approach, as they provide only one picture from the complex disease history, limited by the choice of the landmark (starting) time. If this is fixed close to time 0, the tandem transplant groups are not complete (many of their cases are still classified within the single auto-HCT group), while if it is set later the population is auto-selected, having lost the patients with worst outcome. In particular the tandem auto-allo-HCT group shows an advantage that in part is due to the removal of all patients who died early from TRM.

A solution is represented by combining a series of landmark analyses obtained by moving the landmark time along an interval (we used time from 0 to 3 years) into one model ("supermodel"), and using the latter to compute the survival probabilities of interest according to the pattern of intermediate events occurred[9]. For example, the OS dynamic prediction curves (**Figure 6,** left panel) show the evolution of the predicted probability of surviving for the next 8 years for survivors at all "landmark" time points between 0 and 3 years; each curve corresponds to a different transplant history.

This methodology requires again data preparation steps that can be accomplished having a reasonable expertise, and the use of standard software for Cox regression in the counting processes formulation that can implement "sandwich" variance estimation. Furthermore, it requires programming for the estimation of the conditional probabilities. The difficulty of this step increases with the complexity of time-varying effects. A key part of the application is the choice of the prediction interval (the range of landmark points) and of the time horizon. The time horizon should be clinically relevant, for example in our current study on MM we aimed at predictions for the long term OS and PFS. Also the range of landmark times has to be wide enough to cover the interval where intermediate events occur (in this study, 0-9 months) but also to be able to appreciate the variation of their effects; for example for auto-allo-HCT we aimed to incorporate not only the steep increase of risk at the beginning, but also the beneficial effect in the long term, thus we wanted to have predictions from landmark times at least beyond 2 years. However, in general it might be not feasible to choose large values for both the time horizon and the largest landmark time, because it is necessary to have sufficient information at the maximum time analyzed, which is equal to the sum of the two quantities.

We fixed the range of prediction times equal to (0, 3 years) and the horizon times for OS and PFS roughly corresponding to the survival times reached by 40% of patients: 8 years for OR and 3 years for PFS. Thus for OS the maximum time analyzed was 3+8=11 years, with 991 cases still at risk in total (105 having got auto-allo-HCT); for PFS the maximum time was 3+3=6 years, with 2441 cases still at risk (198 having got auto-allo-HCT).

Multi-variable modelling details

In both the Cox and Poisson modeling approaches, Proportional Hazards (PH) were assumed for the effects of adjustment covariates measured at auto-HCT1. We considered gender, age, disease status at time of transplant, calendar year and interval from diagnosis to auto-HCT1. These variables had no missing values. The selection was done within the application of Cox regression, checking the shape of effects and the PH assumption by analyses of model's residuals. The variables selected were age and calendar year (as continuous variables) and disease status at auto-HCT1, dichotomized as either being in complete remission (CR; baseline) or not being in CR. The Cox models were re-assessed adding a random effect ("frailty") at the center level, to account for possible correlation between observations. The estimated Hazard Ratios and confidence intervals had minimal variation, thus the frailty terms were disregarded.

Supplementary Material References

1 Andersen PK, Pohar Perme M. Inference for outcome probabilities in multi-state models. *Lifetime Data Anal* 2008; **14**: 405–431.

2 De Wreede LC, Fiocco M, Putter H. The mstate package for estimation and prediction in non- and semi-parametric multi-state and competing risks models. *Comput Methods Programs Biomed* 2010; **99**: 261–274.

3 Rebora P, Salim A, Reilly M. bshazard: A Flexible Tool for Nonparametric Smoothing of the Hazard Function. *R J* 2014; **6**: 114.

4 Efron B. The Two-Way Proportional Hazards Model. *J R Stat Soc Ser B Stat Methodol* 2002; **64**: 899–909.

5 Efron B. Logistic Regression, Survival Analysis, and the Kaplan-Meier Curve. *J Am Stat Assoc* 1988; **83**: 414–425.

6 Plummer M, Carstensen B. Lexis : An *R* Class for Epidemiological Studies with Long-Term Follow-Up. *J Stat Softw* 2011; **38**. doi:10.18637/jss.v038.i05.

7 Batyrbekova N, Bower H, Dickman PW, Ravn Landtblom A, Hultcrantz M, Szulkin R *et al.* Modelling multiple time-scales with flexible parametric survival models. *BMC Med Res Methodol* 2022; **22**: 290.

8 Rebora, P., Galimberti, S., & Valsecchi, M. G.. Using multiple timescale models for the evaluation of a time-dependent treatment. *Statistics in Medicine* 2015; **34**: 3648–3660.

9 Van Houwelingen HC, Putter H. Dynamic predicting by landmarking as an alternative for multi-state modeling: an application to acute lymphoid leukemia data. *Lifetime Data Anal* 2008; **14**: 447.
